# Supplementary material for: Exposure to (Poly)phenol Metabolites after a Fruit and Vegetable Supplement Intake: A Double-Blind, Cross-Over, Randomized Trial
Source: Nutrients. 2022 Nov 20;14(22):4913. doi: 10.3390/nu14224913 (PMC9692523; doi:10.3390/nu14224913)
Supplement: Supplementary file 1 [file nutrients-14-04913-s001.zip › nutrients-2014497-supplementary.pdf]

Supplemental Material

Exposure to (poly)phenol metabolites after a fruit and vegetable supplement intake: attempting to mimic the 5-a-day (poly)phenol variety – A double-blind, cross-over, randomized trial

Cindy Romain [1], Letizia Bresciani [2,*], Jananee Muralidharan [1], Pedro Mena [2], Linda H. Chung[3], Pedro E. Alcaraz[3], Daniele Del Rio [2], Julien Cases [1,*]

[1]Innovation and Scientific Affairs, Fytexia, 34350 Vendres, France
[2]Human Nutrition Unit, Department of Food & Drug, University of Parma, Via Volturno 39, 43125 Parma, Italy
[3] Research Center for High Performance Sport - UCAM Universidad Católica de Murcia – Murcia – Spain
*Correspondence: L.B.: letizia.bresciani@unipr.it; Tel.: +39 0521 903906; J.C.: jcases@fytexia.com; Tel: +33 0467 219098

Chemicals and reagents

For powder quantification, punicalin α/A and punicalin β/B mixture, HHDP-gallagyl-hexoside (punicalagin α), ellagic acid, (+)-catechin, (−)-epicatechin, (−)-epicatechin gallate, (−)-epigallocatechin gallate, apigenin 8-*C*-glucoside (vitexin), luteolin-*O*-hexoside, apigenin-7-*O*-rhamnoglucoside, quercetin, isorhamnetin, quercetin-*O*-rutinoside (rutin), luteolin-8-*C* glucoside (orientin), 3-hydroxybenzoic acid, 3,4-dihydroxybenzoic acid (protocatechuic acid), 3,4,5-trihydroxybenzoic acid (gallic acid), 5-caffeoylquinic acid (chlorogenic acid), hydroxytyrosol, oleuropein, verbascoside (caffeoyl phenylethanoid glycoside), naringenin, naringenin-7-*O*-rutinoside (narirutin), hesperetin-7-*O*-rutinoside (hesperidin), isosakuranetin-*O*-rutinoside (didymin), cyanidin-3-*O*-hexoside and cyanidin-3-*O*-rutinoside were purchased from Merck KGaA (Darmstadt, Germany). Procyanidin B2 and piceid (resveratrol-*O*-glucoside) were purchased from PhytoLab (GmbH & Co. KG, Vestenbergsgreuth, Germany).

For metabolite and catabolite quantification, 3′-methoxycinnamic acid-4′-sulfate, 3′-methoxycinnamic acid-4′-glucuronide, 4′-methoxycinnamic acid-3′-sulfate, 4′-methoxycinnamic acid-3′-glucuronide, 3-(3′-methoxyphenyl)propanoic acid-4′-sulfate, 3-(4′-methoxyphenyl)propanoic acid-3′-glucuronide, 3-(4′-hydroxyphenyl)propanoic acid-3′-sulfate, 3-(4′-hydroxyphenyl)propanoic acid-3′-glucuronide, 3′-hydroxycinnamic acid-4′-glucuronide and 4′-hydroxycinnamic acid-3′-glucuronide were purchased from Toronto Research Chemicals (North York, Ontario, Canada). Prof. Alan Crozier (University of California Davis) kindly supplied feruloylglycine. 3-Methyoxybenzoic acid-4-glucuronide, 3-methoxybenzoic acid-4-sulfate, 4-methoxybenzoic acid-3-glucuronide, 4-hydroxybenzoic acid-3-sulfate, 4-hydroxybenzoic acid-3-glucuronide, benzoic acid-4-sulfate, benzoic acid-4-glucuronide were provided by Dr. Colin Kay (N.C. State University's Plants for Human Health Institute). 5-Phenyl-γ-valerolactone-3′-glucuronide, 5-phenyl-γ-valerolactone-3′-sulfate, 5-(4′-hydroxyphenyl)-γ-valerolactone-3′-sulfate, 5-(5′-hydroxyphenyl)-γ-valerolactone-3′-glucuronide were synthesized in house [1,2]. HPLC-grade solvents were purchased from VWR International (Radnor, PA, USA). Ultrapure water from MilliQ system (Millipore, Bedford, MA, USA) was used throughout the experiment.

References

[1]. Curti, C., N. Brindani, L. Battistini, A. Sartori, G. Pelosi, P. Mena, F. Brighenti, F. Zanardi and D. Del Rio (2015). "Catalytic, Enantioselective Vinylogous Mukaiyama Aldol Reaction of Furan-Based Dienoxy Silanes: A Chemodivergent Approach to γ-Valerolactone Flavan-3-ol Metabolites and δ-Lactone Analogues." Advanced Synthesis & Catalysis 357(18): 4082-4092.

[2]. Brindani, N., P. Mena, L. Calani, I. Benzie, S. W. Choi, F. Brighenti, F. Zanardi, C. Curti and D. Del Rio (2017). "Synthetic and analytical strategies for the quantification of phenyl-gamma-valerolactone conjugated metabolites in human urine." Mol Nutr Food Res 61(9).

Supplemental Table S1. Retention time (RT), spectrometric characteristics and standards used for quantification of detected native (poly)phenols occurring in the supplement.

Compound	RT (min)	[M-H]-	MS²	MS³	Standard
**Ellagitannins**					
Ellagic acid	6.35	301	301, 257, 229, 185, 151, 121		Ellagic acid
Galloyl-hexoside	0.99	331	169, 271, 211, 241, 193, 125	125	Gallagyl-hexoside (Punicalin α/A and Punicalin β/B)
Galloyl-hexoside	1.32	331	169, 271, 211, 241, 193, 125	125	Gallagyl-hexoside (Punicalin α/A and Punicalin β/B)
Galloyl-hexoside	1.66	331	169, 271, 211, 241, 193, 125	125	Gallagyl-hexoside (Punicalin α/A and Punicalin β/B)
Galloyl-hexoside	2.00	331	271, 241, 169	125	Gallagyl-hexoside (Punicalin α/A and Punicalin β/B)
Galloyl-hexoside	2.34	331	271, 241, 169	125	Gallagyl-hexoside (Punicalin α/A and Punicalin β/B)
Ellagic acid hexoside	5.37	463	301, 300, 302	257, 301, 229, 284, 185, 300, 255, 213	Gallagyl-hexoside (Punicalin α/A and Punicalin β/B)
Ellagic acid dimethyl ether glucurionide	6.31	507	331, 489		Gallagyl-hexoside (Punicalin α/A and Punicalin β/B)
Galloyl-HHDP-hexoside	2.01	633	301, 249, 481, 564, 624, 275, 463	301, 257, 284, 229, 157	Gallagyl-hexoside (Punicalin α/A and Punicalin β/B)
Galloyl-HHDP-hexoside	2.38	633	301, 481, 249, 275, 565, 610	257, 185	Gallagyl-hexoside (Punicalin α/A and Punicalin β/B)
Galloyl-HHDP-hexoside	2.75	633	615, 301, 481, 419, 275	571, 419, 299, 343, 329, 287	Gallagyl-hexoside (Punicalin α/A and Punicalin β/B)
Galloyl-HHDP-hexoside	3.49	633	301, 615, 431, 275, 481		Gallagyl-hexoside (Punicalin α/A and Punicalin β/B)
Galloyl-HHDP-hexoside	3.83	633	301, 421, 275, 451, 615, 589		Gallagyl-hexoside (Punicalin α/A and Punicalin β/B)
Galloyl-HHDP-hexoside	4.46	633	301, 275	257, 301, 229, 284, 273, 185	Gallagyl-hexoside (Punicalin α/A and Punicalin β/B)
Galloyl-HHDP-hexoside	4.81	633	301, 463, 275	257, 284, 229, 185, 301, 200, 258, 285, 173	Gallagyl-hexoside (Punicalin α/A and Punicalin β/B)
Gallagyl-hexoside (Punicalin α/A)	2.21	781	601, 721, 575	299, 271	Gallagyl-hexoside (Punicalin α/A and Punicalin β/B)
Gallagyl-hexoside (Punicalin β/B)	2.62	781	601, 721, 575		Gallagyl-hexoside (Punicalin α/A and Punicalin β/B)
Bis-HHDP-hexoside (Pedunculagin I isomer)	3.32	783	301, 481, 765	257, 229, 301	Gallagyl-hexoside (Punicalin α/A and Punicalin β/B)
Bis-HHDP-hexoside (Pedunculagin I isomer)	3.68	783	301, 481, 765	257, 229, 301	Gallagyl-hexoside (Punicalin α/A and Punicalin β/B)
Bis-HHDP-hexoside	4.01	783	301, 481, 765	257, 229, 301	Gallagyl-hexoside (Punicalin α/A and Punicalin β/B)

(Pedunculagin I isomer)					
Galloyl-bis-HHDP-hexoside (Casuarinin)	4.64	935	633, 917, 873, 659	571, 589, 615, 299, 481	HHDP-gallagyl-hexoside (Punicalagin α)
Punicalagin isomer	3.29	1083	807, 601, 1021, 721, 959, 575		HHDP-gallagyl-hexoside (Punicalagin α)
HHDP-gallagyl-hexoside (Punicalagin α)	3.97	1083	601, 781, 575	299, 271, 243	HHDP-gallagyl-hexoside (Punicalagin α)
HHDP-gallagyl-hexoside (Punicalagin β)	4.36	1083	601, 781, 575	299, 271, 243	HHDP-gallagyl-hexoside (Punicalagin β)
Di(HHDP-galloylglucose)-pentoside	3.41	707 $[M-2H]^{2-}$	783, 613, 633, 1113, 933	301, 721, 481, 765, 421, 275, 703	HHDP-gallagyl-hexoside (Punicalagin β)
Di(HHDP-galloylglucose)-pentoside	3.77	707 $[M-2H]^{2-}$	783, 613, 633, 1113, 933	301, 721, 481, 765, 421, 275, 703	HHDP-gallagyl-hexoside (Punicalagin β)
Di(HHDP-galloylglucose)-pentoside	4.13	707 $[M-2H]^{2-}$	783, 613, 633, 1113, 933	301, 721, 481, 765, 421, 275, 703	HHDP-gallagyl-hexoside (Punicalagin β)
**Gallotannins**					
Digalloylglucose	4.44	483	271, 331, 313, 272	211, 169	Gallagyl-hexoside (Punicalin α/A and Punicalin β/B)
Digalloylglucose	2.40	483	331, 313, 169		Gallagyl-hexoside (Punicalin α/A and Punicalin β/B)
Digalloylglucose	3.47	483	331, 313, 169		Gallagyl-hexoside (Punicalin α/A and Punicalin β/B)
Digalloylglucose	3.94	483	423, 313, 271, 331, 193, 241,169		Gallagyl-hexoside (Punicalin α/A and Punicalin β/B)
Digalloylglucose	4.28	483	423, 271, 313, 169, 465		Gallagyl-hexoside (Punicalin α/A and Punicalin β/B)
Trigalloylglucose	5.30	635	465, 483, 466, 313, 484	313, 169, 295	Gallagyl-hexoside (Punicalin α/A and Punicalin β/B)
**Dihydrochalcones**					
Phloretin	8.73	273	167	123, 125, 151	(+)-Catechin
**Flavan-3-ols**					
(+)-Catechin	4.53	289	245, 205, 179, 125		(+)-Catechin
(−)-Epicatechin	5.06	289	245, 205, 179, 125		(−)-Epicatechin
(+)-Gallocatechin	3.51	305	179, 221, 219, 261, 165, 125		(−)-Epicatechin
(−)-Epigallocatechin	4.28	305	179, 221, 219, 261, 165, 125		(−)-Epicatechin
(Epi)catechin gallate	5.97	441	289, 169, 331, 271, 397, 303, 193, 243		(-)-Epicatechin gallate
(Epi)gallocatechin-methylgallate	5.84	471	183, 305, 287, 168, 269		(-)-Epicatechin gallate

(−)-Epigallocatechin gallate	5.16	457	169, 331, 305, 269, 193, 287		(-)-Epigallocatechin gallate
(Epi)gallocatechin gallate	5.50	457	169, 331, 305, 287, 193, 269		(-)-Epigallocatechin gallate
Procyanidin dimer A-type	5.78	575	449, 423, 289, 285, 539, 557, 407		Procyanidin B2
Procyanidin dimer B-type	4.23	577	425, 407, 451, 289, 559, 245		Procyanidin B2
Procyanidin dimer B-type	4.73	577	425, 407, 451, 289, 559, 245		Procyanidin B2
Procyanidin dimer B-type	4.47	577	425, 407, 451, 289, 559, 246		Procyanidin B2
Procyanidin dimer B-type	5.47	577	425, 407, 451, 289, 559, 247		Procyanidin B2
Prodelphinidin dimer B-type [1 unit of (epi)GC+1(epi)C]	3.70	593	549, 575, 467, 423, 289, 305, 440		Procyanidin B2
Prodelphinidin dimer B-type [1 unit of (epi)GC+1(epi)C]	4.39	593	549, 575, 467, 423, 289, 305, 441		Procyanidin B2
Prodelphinidin dimer B-type gallate [1 unit of (epi)GC+1(epi)C]	4.89	745	593, 423, 575, 619, 577, 467, 559, 407, 305, 727, 457, 289, 331		Procyanidin B2
Prodelphinidin tetramer B-type gallate [2 unit of (epi)GC+2(epi)C]	4.06	745 [M-2H]2-	701, 575, 601		Procyanidin B2
Prodelphinidin tetramer B-type gallate [2 unit of (epi)GC+2(epi)C]	4.46	745 [M-2H]2-	701, 575, 593		Procyanidin B2
Prodelphinidin dimer B-type gallate [2 units (epi)GC]	6.78	761	609, 686, 563, 738, 693, 439, 599, 348, 744, 256		Procyanidin B2
Procyanidin trimer B-type	4.50	865	695, 575, 577, 713, 739, 847, 543, 287		
Procyanidin trimer B-type	4.86	865	695, 739, 713, 577, 575, 780, 847, 425, 587, 801, 287		
Procyanidin trimer B-type	5.29	865	695, 712, 577, 739, 407, 425, 287		
(Epi)catechin-gallate dimer	6.03	885	441, 442, 443, 289		
**Flavones**					

Apigenin	9.24	269	225, 149		
Trihydroxyflavone	5.23	269	225, 227, 241, 197, 182, 251		Naringenin
Apigenin-*O*-hexoside	6.88	431	269		Apigenin 8-C-glucoside (Vitexin)
Luteolin-*O*-hexoside	4.67	447	285	241, 243, 199, 175, 257, 217	Luteolin-*O*-hexoside
Luteolin-*O*-hexoside	6.28	447	285	241, 243, 199, 175, 257, 217	Luteolin-*O*-hexoside
Luteolin-*O*-hexoside	6.67	447	285	241, 243, 199, 175, 257, 217	Luteolin-*O*-hexoside
Luteolin-*O*-hexoside	7.02	447	285	241, 243, 199, 175, 257, 217	Luteolin-*O*-hexoside
Dihydroluteolin-*O*-hexoside	5.25	449	287	243,125,161,269	Luteolin-*O*-hexoside
Apigenin-*O*-rutinoside	6.63	577	269		Apigenin-7-*O*-rhamnoglucoside
Luteolin-*O*-rutinoside	6.07	593	285	241, 217, 243, 199, 175, 257	Apigenin-7-*O*-rhamnoglucoside
Luteolin-*O*-rutinoside	8.10	593	285	241, 217, 243, 199, 175, 257	Apigenin-7-*O*-rhamnoglucoside
Chrysoeriol-*O*-rutinoside or Diosmetin-*O*-rutinoside	6.85	607	299, 284	284	Apigenin-7-*O*-rhamnoglucoside
Luteolin-*O*-dihexoside (tentative identification)	5.45	609	447, 285		Apigenin-7-*O*-rhamnoglucoside
Luteolin-*O*-dihexoside	7.42	609	285, 447	241,243,217,199,175,257	Apigenin-7-*O*-rhamnoglucoside
**Flavonols**					
Quercetin	8.52	301	179, 151		Quercetin
Rhamnetin	8.63	315	300, 301		Isorhamnetin
Isorhamnetin	9.56	315	300, 301		Isorhamnetin
Myricetin	7.17	317	179, 151		Isorhamnetin
Quercetin-*O*-pentoside	6.61	433	301, 300	179,151,257	Quercetin-*O*-rutinoside (Rutin)
Myricetin-*O*-rhamnoside	6.12	463	301, 300, 316, 317		Luteolin-*O*-hexoside
Quercetin-*O*-hexoside	6.24	463	301, 300, 302	179,151,257	Quercetin-*O*-rutinoside (Rutin)
Quercetin-*O*-glucuronide	6.33	477	301	179, 151, 257, 273	Quercetin-*O*-rutinoside (Rutin)
Myricetin-*O*-hexoside	5.72	479	316, 317, 179, 461	271, 179, 287, 270, 288, 151, 242, 272	Luteolin-*O*-hexoside
Kaempferol-*O*-rutinoside	6.5	593	285	257,267,299,241,197	Quercetin-*O*-rutinoside (Rutin)
Tetrahydroxy-dimethoxyflavone-*O*-hexoside (Syringetin-*O*-hexoside)	6.75	507	345, 344, 461, 387, 201, 293, 417, 329, 439, 489	330, 301	Luteolin-*O*-hexoside
Isorhamnetin-*O*-dirhamnoside	6.56	607	461	315, 135, 161	Luteolin-*O*-hexoside
Quercetin-*O*-rutinoside (Rutin)	6.02	609	301	179, 151, 257	Quercetin-*O*-rutinoside (Rutin)
Isorhamnetin-*O*-rutinoside	6.54	623	315		Luteolin-*O*-hexoside
**Flavanones**					

Naringenin	8.79	271	151, 177		Naringenin
Eriodyctiol	7.89	287	151		Naringenin
Tetrahydroxyflavanone	10.03	287	269, 241, 219, 113		Naringenin
Naringenin-*O*-glucoside	5.19	433	271	151, 177	Naringenin-7-*O*-rutinoside (Narirutin)
Tetrahydroxyflavanone-*O*-rhamnoside(tentative identification)	6.93	433	269, 287, 259, 301		Naringenin-7-*O*-rutinoside (Narirutin)
Hesperetin-*O*-hexoside	7.06	463	301	179, 151, 286, 257	Hesperetin-7-*O*-rutinoside (Hesperidin)
Naringenin-7-*O*-rutinoside (Narirutin)	6.29	579	271	151, 177	Naringenin-7-*O*-rutinoside (Narirutin)
Naringenin-7-*O*-neohesperidoside(Naringin)	7.03	579	459, 541, 271, 235	357, 339, 235, 271, 441	Naringenin-7-*O*-rutinoside (Narirutin)
Naringenin-*O*-neohesperidoside	7.11	579	459, 541, 271, 235		Naringenin-7-*O*-rutinoside (Narirutin)
Isosakuranetin-*O*-rutinoside (Didymin)	7.60	593	285	270, 243, 164, 151, 241	Isosakuranetin-O-rutinoside (Didymin)
Eriocitrin or Neoeriocitrin	5.92	595	459	357, 441, 235, 271	*
Naringenin-*C*-dihexoside	5.93	595	271, 475		Luteolin-8-*C* glucoside (Orientin)
Hesperetin-7-*O*-rutinoside (Hesperidin)	6.64	609	301	286, 283, 242,257	Hesperetin-7-*O*-rutinoside (Hesperidin)
Hesperetin-7-*O*-neohesperidoside (Neohesperidin)	6.76	609	301	286, 283, 242,257	Hesperetin-7-*O*-neohesperidoside(Neohesperidin)
**Hydroxybenzoic acids**					
3-Hydroxybenzoic acid	5.06	137	93		3-Hydroxybenzoic acid
4-Hydroxybenzoic acid	4.64	137	109, 93		3-Hydroxybenzoic acid
Hydroxybenzoic acid	4.31	137	109, 93		3-Hydroxybenzoic acid
3,4-Dihydroxybenzoic acid (Protocatechuic acid)	3.59	153	109		3,4-Dihydroxybenzoic acid (Protocatechuic acid)
Dihydroxybenzoic acid	5.58	153			3,4-Dihydroxybenzoic acid (Protocatechuic acid)
Dihydroxyphenylacetic acid	3.20	167	123, 139, 149		3,4-Dihydroxybenzoic acid (Protocatechuic acid)
Dihydroxyphenylacetic acid	4.36	167	123,139,149,81		3,4-Dihydroxybenzoic acid (Protocatechuic acid)
Dihydroxyphenylacetic acid	5.09	167	123,81,139		3,4-Dihydroxybenzoic acid (Protocatechuic acid)
Gallic acid	2.10	169	125		Gallic acid
Hydroxyphenyllactic acid	3.89	181	163, 113, 135, 151		
Ethyl-gallate	5.68	197	169, 168, 125		Gallic acid
**Hydroxycinnamic acids**					
Caffeic acid	5.14	179	135		3,4-Dihydroxybenzoic acid (Protocatechuic acid)

5-Caffeoylquinic acid	4.75	353	191		5-Caffeoylquinic acid
Ferulic acid-*O*-hexoside	5.14	355	193		Luteolin-*O*-hexoside
**Phenylethanoids**					
Tyrosol (tentative identification)	3.62	137	109,93,122		3-Hydroxybenzoic acid
Hydroxytyrosol	3.37	153	123		Hydroxytyrosol
Hydroxytyrosol-*O*-hexoside	3.20	315	135, 153, 113, 119, 179, 247	123	Hydroxytyrosol
Oleoside	4.25	389	345, 209, 121, 165		Oleuropein
Verbascoside (Caffeoyl phenylethanoid glycoside)	5.95	623	461		Verbascoside (Caffeoyl phenylethanoid glycoside)
Verbascoside (Caffeoyl phenylethanoid glycoside)	6.29	623	461		Verbascoside (Caffeoyl phenylethanoid glycoside)
**Coumarin**					
Scopoletin-*O*-hexoside	4.75	353	191	173, 171, 127, 85	
**Stilbenoid**					
Piceid (Resveratrol-*O*-glucoside)	3.35	389	227, 183, 165, 209, 121, 139		Piceid (Resveratrol-*O*-glucoside)
**Seco-iridoid**					
Oleuropein aglycone	7.52	377	307, 275	275, 139	
Oleuropein aglycone	8.11	377	307, 275	275, 139	
Oleuropein aglycone	8.44	377	307, 275	275, 139	
Oleuropein aglycone	9.04	377	307, 275	275, 139	
Oleuropein aglycone	9.37	377	307, 275	275, 139	
Oleuropein	7.05	539	377, 307, 275	307, 275	Oleuropein
Oleuropein	7.39	539	377, 307, 275	307, 275	Oleuropein
**Anthocyanins**					
Cyanidin-3-*O*-hexoside	4.70	449	287	287, 153	Cyanidin-3-*O*-hexoside
Malvidin-3-*O*-arabinoside	5.42	463	331	316, 315, 299, 298, 287, 270, 179, 242, 331	Cyanidin-3-*O*-hexoside
Peonidin-3-*O*-hexoside	5.15	463	301		Cyanidin-3-*O*-hexoside
Delphinidin 3-*O*-hexoside	4.50	465	303		Cyanidin-3-*O*-hexoside
Petunidin-3-*O*-hexoside	4.86	479	317	302	Cyanidin-3-*O*-hexoside
Malvidin-3-*O*-hexoside	5.21	493	331	316, 315, 299, 298, 287, 270, 179, 242, 331	Cyanidin-3-*O*-hexoside

Cyanidin-3-*O*-rutinoside	4.86	595	287	Cyanidin-3-*O*-rutinoside
Delphinidin 3-*O*-rutinoside	4.60	611	303, 465, 287	Cyanidin-3-*O*-rutinoside
**TOTAL ((POLY))PHENOL**				

HHDP: means hexahydroxydiphenoyl ; (epi)GC means (epi)gallocatechin ; (epi)C means (epi)catechin; n.q means compounds which have been identified but not quantified because <LOQ.

Supplemental Table S2. Spectrometric characteristics of quantified metabolites in plasma and urine samples. *m/z*: mass to charge ratio, RT: retention time; CE: collision energy.

Id.	Phenolic metabolites	Parent ion (*m/z*)	RT (min)	S-lens	Quantifier ion Product ion (m/z)	CE (V)
	**(Epi)catechin derivatives**					
1	Methoxy-(epi)catechin-glucuronide	479	3.47	92	303	35
2	(Epi)catechin-glucuronide_isomer 1	465	3.83	98	289	27
3	Methoxy-(epi)gallocatechin-glucuronide	495	3.89	98	319	27
4	(Epi)catechin-glucuronide_isomer 2	465	4.21	98	289	27
5	(Epi)catechin-sulfate_isomer 1	369	4.44	93	289	20
6	(Epi)catechin-sulfate_isomer 2	369	4.73	93	289	20
7	Methoxy(epi)catechin-sulfate	383	4.97	92	303	20
	**Phenyl-y-valerolactones & phenylvaleric acids**					
8	5-(Dihydroxyphenyl)-γ-valerolactone-glucuronide	399	2.00	87	223	30
9	5-(Methoxyhydroxyphenyl)-γ-valerolactone- glucuronide	413	2.03	87	222	30
10	5-(5@Hydroxyphenyl)-γ-valerolactone-3@glucuronide	383	2.32	92	207	27
11	4-Hydroxy-5-(methoxyphenyl)valeric acid-glucuronide (4-Hydroxy-5-phenylvaleric acid-methoxy-glucuronide)	415	3.56	93	239	30
12	5-(Methoxyphenylvaleric) acid-sulfate	303	3.65	80	223	20
13	5-(Phenyl)-γ-valerolactone-sulfate-glucuronide	463	3.83	87	287	27
14	5-(Methoxy-phenylvaleric) acid--glucuronide	399	3.99	80	223	30
15	4-Hydroxy-5-(Hydroxyphenyl)valeric acid-sulfate	305	4.00	95	225	20
16	5-(5@Hydroxyphenyl)-γ-valerolactone-3@sulfate	287	4.14	96	207	23
17	5-(Phenyl)-γ-valerolactone-3@glucuronide	367	4.30	93	191	25
18	4-Hydroxy-5-(phenyl)valeric acid-sulfate	289	4.32	95	209	20
19	4-Hydroxy-5-(methoxy-phenyl)valeric acid--sulfate	319	4.35	63	224	30
20	5-(Methoxy-hydroxyphenyl)-γ-valerolactone--sulfate	317	4.38	97	222	40
21	5-(3@Hydroxyphenyl)-γ-valerolactone-4@sulfate	287	4.70	96	207	23
22	5-(Methoxy-phenyl)-γ-valerolactone-sulfate	301	4.88	96	221	23
23	5-(Phenyl)-γ-valerolactone-3@sulfate	271	5.02	93	191	23
24	5-(Hydroxyphenyl)valeric acid-sulfate	289	5.56	95	209	20
	**Flavanone derivatives**					
25	Naringenin-diglucuronide	623	4.51	100	447	25
26	Hesperetin-diglucuronide	653	4.80	115	301	26
27	Naringenin-glucuronide	447	5.15	100	271	26
28	Hesperetin-7-glucuronide	477	5.27	115	301	27

29	Hesperetin-sulfate	381	6.61	84	301	24
	**Other flavonoid derivatives**					
30	Luteolin-glucuronide	461	5.78	90	285	27
31	Myricetin-glucuronide	493	4.01	90	317	27
32	Quercetin-diglucuronide	653	4.30	115	301	26
48	Luteolin-sulfate	365	6.19	90	285	20
	**Phenylethanoid derivatives**					
33	2-(Phenyl)ethanol-3′-glucuronide (Hydroxytyrosol-glucuronide)	329	1.55	64	153	20
34	Oleuropein-sulfate	619	5.05	100	539	20
	**Hydroxybenzoic acids and simple benzenes**					
35	4-Hydroxybenzoic acid-3-glucuronide (Protocatechuic acid-3-glucuronide)	329	1.44	81	153	21
36	Dihydroxybenzene-sulfate (Pyrogallol-sulfate)	205	2.40	68	125	20
37	Methoxyhydroxybenzene-sulfate (Pyrogallol-methoxy-sulfate)	219	3.78	68	124	24
38	Methoxy-hydroxybenzoic acid-sulfate (Gallic acid-methoxy-sulfate)	263	2.96	68	168	26
39	3,5-Dimethoxy-4-hydroxybenzoic acid (Syringic acid)	197	4.24	70	182	20
45	4-Hydroxybenzoic acid-3-sulfate (Protocatechuic acid-3-sulfate)	233	2.42	85	153	20
47	4′-Hydroxyhippuric acid	194	1.50	72	100	11
	**(Hydroxyphenyl)propanoic acids**					
40	3-(3′-Hydroxyphenyl)propanoic acid (3-(3-Hydroxyphenyl)propionic acid)	165	3.25	48	121	13
41	3-(4′-hydroxyphenyl)propanoic acid-3′-sulfate (Dihydrocaffeic acid-sulfate)	261	4.04	96	181	20
46	3-(3′-Methoxyphenyl)propanoic acid-4′-sulfate (Dihydroferulic acid-sulfate)	275	4.39	75	195	31
	**Ellagitannin derivatives**					
42	8-Hydroxy-urolithin-3-glucuronide	307	7.10	78	227	19

	(Urolithin A-glucuronide)					
43	9-Hydroxy-urolithin-3-glucuronide*	307	7.03	78	227	19
	(Isourolithin A-glucuronide)					
44	Urolithin-3-glucuronide	387	5.24	78	211	37
	(Urolithin B-glucuronide)					

* Qualifier ion (*m*/*z*) = 171
